# Skin prick test reactivity to common allergens among women in Entebbe, Uganda

**DOI:** 10.1016/j.trstmh.2008.01.017

**Published:** 2008-04

**Authors:** Harriet Mpairwe, Lawrence Muhangi, Juliet Ndibazza, Josephine Tumusiime, Moses Muwanga, Laura C. Rodrigues, Alison M. Elliott

**Affiliations:** a MRC/UVRI Uganda Research Unit on AIDS, Uganda Virus Research Institute, P.O. Box 49, Entebbe, Uganda; b Entebbe Hospital, P.O. Box 29, Entebbe, Uganda; c London School of Hygiene & Tropical Medicine, Keppel Street, London, WC1E 7HT, UK

**Keywords:** Worms, Allergy, Atopy, Skin prick test, *Mansonella perstans*, Uganda

## Abstract

The objectives of this study were to estimate the prevalence of atopic sensitization, and to identify common aeroallergens associated with atopic sensitization among women in Entebbe, Uganda, and to determine risk factors for atopic sensitization among those with and without a history of asthma or eczema. A case–control study was conducted within a trial of deworming in pregnancy, approximately 2 years after the intervention. Skin prick test reactivity was assessed among 20 women with a history of asthma, 25 with history of eczema and 95 controls. Overall prevalence of reactivity was estimated by adjusting for the prevalence of asthma in the whole cohort. Overall skin prick test prevalence was: any allergen 30.7%, *Blomia tropicalis* 10.9%, *Dermatophagoides* mix 16.8%, cockroach 15.8%. The prevalence of a positive skin prick test was significantly associated with a history of asthma (70% to any allergen vs. 32%, *P* = 0.002) but not with a history of eczema (44% vs. 36%, *P* = 0.49). Women with *Mansonella perstans* had significantly reduced odds for atopic sensitization (adjusted odds ratio 0.14, 95% CI 0.03–0.69); women with a history of asthma were less likely to have hookworm (adjusted odds ratio 0.24, 95% CI 0.07–0.81) but this association was weaker for women with a history of eczema. [Clinical Trial No. ISRCTN32849447]

## 1 Introduction

Allergic diseases are on the rise worldwide, and in developing countries the prevalence is higher in urban than in rural areas (Keeley et al., 1991, Ng’ang’a et al., 1998, Van Niekerk et al., 1979, Yemaneberhan et al., 1997). A number of factors related to lifestyle and environmental exposures may explain these observations. Among these, it has been proposed that increases in allergic disease may be related to the immunological effects of a decline in infectious diseases in more developed and urban environments. In particular, several epidemiological studies have shown an inverse relationship between chronic worm infections and allergy (Cooper et al., 2003, Nyan et al., 2001; van den Biggelaar et al., 2000). Further, immunological studies have suggested that the immune-modulating effects of chronic worm infection can be transferred to a fetus in utero (Malhotra et al., 1997). In a preliminary study of the effects of worm infections in pregnancy among 104 women and their infants, we found an inverse association between maternal worm infections and incidence of infantile eczema, and a possibility that the incidence of infantile eczema might be increased by deworming of women with albendazole during pregnancy (Elliott et al., 2005). We plan to explore this further in our ongoing trial of deworming among 2500 pregnant women in Entebbe, Uganda [ISRCTN32849447] (Elliott et al., 2007), through further studies both of skin prick test responses and of allergic disease incidence in infancy and childhood.

Skin prick testing is a useful way of establishing what allergens a person is sensitized to, but it is important to use allergens relevant to the person's environment as the sensitization pattern may differ across regions. Prior to this study, for Uganda in general and Entebbe in particular, no such investigation had ever been carried out.

The objectives of this study were to estimate the prevalence of atopic sensitization, and to identify the common aeroallergens associated with atopic sensitization and the risk factors for atopic sensitization among women with a history of asthma or eczema, and among women with no such history in Entebbe, Uganda.

## 2 Methods

### 2.1 Study population

The study participants were recruited from women in an ongoing trial, the Entebbe Mother and Baby Study (EMABS), in Entebbe and Katabi, a peri-urban area along the shores of Lake Victoria in Uganda. Details of the EMABS trial have been described (Elliott et al., 2007). In summary, pregnant women were recruited from the Entebbe hospital antenatal clinic, a free government facility, during their second or third trimester. Women who fulfilled the trial inclusion criteria and were interested in the study gave written informed consent and were requested to provide a stool sample. Blood samples were also drawn for routine tests (such as haemoglobin level and syphilis serology), for HIV serology, and for examination for malaria parasites and *Mansonella perstans* (the only common filarial worm in the study area). The women were then enrolled in a double-blind, placebo-controlled trial of treatment of maternal worms in pregnancy with albendazole versus placebo and praziquantel versus placebo in a 2 × 2 factorial design. As part of standard antenatal care at the clinic, all women received intermittent presumptive treatment for malaria with sulfadoxine–pyrimethamine, and HIV-positive women were enrolled in a programme for prevention of mother-to-child HIV transmission using nevirapine. Following delivery, women provided another stool and blood sample and, at 6 weeks post delivery, they all received albendazole and praziquantel. Children continued to attend the study clinic for routine follow up for immunizations in infancy and for quarterly study visits thereafter.

An additional questionnaire regarding maternal and family history of allergic disease was administered to mothers attending with their children when the children were 1 year old.

### 2.2 Study design

Women whose children had reached 1 year or more, and who had responded to the questionnaire on allergic disease, were eligible for recruitment to this study in an unmatched case–control study design. The sample included, as cases, all mothers reporting a history of asthma and or eczema and, as controls, a random sample selected from those who had no such history. The participants gave free and informed written consent.

### 2.3 Skin prick testing

Skin prick testing of the women was conducted at least 1 year after delivery (and at least 10 months after treatment with both albendazole and praziquantel). Tests were performed using standardized extracts from organisms known to be present in the study environment (ALK Abello, Hoersholm, Denmark): *Blomia tropicalis*; *Dermatophagoides* mix (*D. farinae*, *D. pteronyssinus*); *Cynodon dactylon* (Bermuda grass); pollen mix (*Artemisia*, *Chenopodium*, *Parietaria*, *Plantago*); mould mix (*Alternaria*, *Chaetomium*, *Cladosporium fulvum*, *Cladosporium herbarum*, *Fusarium*); cat (*Felix domesticus*) and dog (*Canis familiaris*) epithelia; and American cockroach (*Periplaneta americana*). Histamine was used as a positive control and saline solution as the negative control. A mean wheal diameter of at least 3 mm greater than the negative control was taken as positive, with the reading taken after 15 min.

### 2.4 Reported history of asthma and or eczema

Women were scheduled to visit the study clinic when their children were 1 year old. During the visit, the women were asked if they themselves had ever had asthma and or eczema. Asthma has an equivalent term in the local language but eczema does not, so it was described as a recurrent itchy rash associated with a dry or weeping skin affecting predominantly flexures (inside the elbows, behind the knees) and frictional areas such as neck, wrists and ankles, as well as below the buttocks, in the armpits, and around the eyes and ears.

### 2.5 Statistical methods

We estimated that a sample of 56 cases and 112 controls would have 80% power with 0.05 significance level to detect a difference of 30% versus 10% positive skin prick test among cases and controls respectively, a plausible difference in the light of results obtained elsewhere. This same sample size would also give 80% power to demonstrate skin prick test reactivity to common allergens of 10% (±3.5%) among the study women.

Data were collected on pre-coded forms and questionnaires and manually checked before double data entry using Microsoft Access (Microsoft Corp., Redmond, WA, USA). Data were analysed using STATA version 8 (Stata Corp., College Station, TX, USA). Prevalence of skin prick test reactivity to each allergen was calculated separately for cases and controls. To obtain a crude estimate of the overall prevalence of skin prick test reactivity in the trial population, and for the study of the risk factors associated with atopy, six randomly selected women with asthma/eczema were added to the controls to create a reconstituted population with the same prevalence of reported frequency of asthma (6%) as that in the general trial population. Initial comparisons in the reconstituted population and between atopic and non-atopic cases and controls were made using simple tables and χ^2^ tests. Logistic regression was used to obtain crude and adjusted odds ratios for the associations between atopy and allergic diseases and helminth infection.

## 3 Results

Recruitment of the 2507 participants for the main trial started in 2002 and was completed in 2005. Prior to treatment, common infections among these participants were hookworm (44.5%), *M. perstans* (21.3%), *Schistosoma mansoni* (18.3%), asymptomatic *Plasmodium falciparum* parasitaemia (10.9%) and HIV (11.9%), as previously reported (Muhangi et al., 2007). By January 2006, 790 babies had attended with their mothers at age 1 year. Of these, 50 mothers had a history of asthma and/or eczema and were selected for this skin prick testing study, together with a random selection of 112 controls. Of mothers selected for the skin prick testing study, 43/50 (86%) of cases and 95/112 (85%) of controls attended and were studied. Skin prick testing among these participants was conducted between February and May 2006.

### 3.1 Prevalence of positive skin prick test to different allergens

Skin prick test results showed that sensitization to house dust mite allergens (*B. tropicalis* and *Dermatophagoides* mix) and to cockroach was most common in this environment (Table 1). Sensitization to the other allergens tested was rare: only three women reacted to *C. dactylon* allergen and three to *F. domesticus* allergen. Only one woman reacted to a pollen mix of common weeds (*Artemisia*, *Chenopodium*, *Parietaria*, *Plantago*), and no woman reacted to the mould mix. No woman reacted to saline, the negative control. The prevalence of positive skin prick test responses was significantly higher among women with a reported history of asthma, than in those with no history of asthma, but the difference between women with and without eczema was not statistically significant (Table 1).

**Table 1:** Skin prick test results of women with and without a history of asthma and eczema in Entebbe, Uganda

Allergen tested		Asthma (*n* = 20) *n* (%)	No asthma (*n* = 117) *n* (%)	Adjusted odds ratio^a^ (95% CI)	*P-*value	Eczema (*n* = 25) *n* (%)	No eczema (*n* = 112) *n* (%)	Adjusted odds ratio^a^ (95% CI)	*P*-value
Any allergen	Negative	6 (30)	80 (68)	1	0.007	14 (56)	72 (64)	1	0.30
	Positive	14 (70)	37 (32)	4.38 (1.50–12.80)		11 (44)	40 (36)	1.62 (0.65–4.05)	

*Blomia tropicalis*	Negative	9 (45)	101 (86)	1	0.001	18 (72)	92 (82)	1	0.19
	Positive	11 (55)	16 (14)	6.03 (2.00–18.24)		7 (28)	20 (18)	2.05 (0.70–5.99)	

*Dermatophagoides* mix^b^	Negative	8 (40)	94 (80)	1	0.003	17 (68)	85 (76)	1	0.31
	Positive	12 (60)	23 (20)	4.99 (1.74–14.32)		8 (32)	27 (24)	1.66 (0.62–4.45)	

American cockroach	Negative	11 (55)	96 (82)	1	0.02	17 (68)	90 (80)	1	0.16
	Positive	9 (45)	21 (18)	3.49 (1.23–9.90)		8 (32)	22 (20)	2.03 (0.75–5.49)	

Dog	Negative	16 (80)	112 (96)	1	0.02	23 (92)	105 (94)	1	0.72
	Positive	4 (20)	5 (4)	6.08 (1.38–26.83)		2 (8)	7 (6)	1.35 (0.26–7.00)	

a Adjusted for age, education and household socioeconomic status.

b D. farinae, D. pteronyssinus.

### 3.2 Characteristics of participants with and without a reported history of allergic disease

Characteristics of participants with and without a reported history of asthma or eczema are presented in Table 2. The age distribution, haemoglobin levels and socioeconomic status were similar for women with a history of asthma or eczema and those with no such history. The education level for the women who reported a history of asthma was higher than that of mothers with eczema or neither history, but these differences were not statistically significant.

**Table 2:** Characteristics of women with a history of asthma or eczema compared to women without a history of asthma or eczema, respectively in Entebbe, Uganda

Characteristic		History of asthma (*n* = 20) *n* (%)	No history of asthma (*n* = 117) *n* (%)	Adjusted odds ratio^a^ (95% CI)	*P-*value	History of eczema (*n* = 25) *n* (%)	No history of eczema (*n* = 112) *n* (%)	Adjusted odds ratio^a^ (95% CI)	*P-*value
Age (years)	14–24	10 (50)	76 (65)	1	0.21	15 (60)	71 (63)	1	0.83
	≥25	10 (50)	41 (35)	1.95 (0.69–5.49)		10 (40)	41 (37)	0.90 (0.35–2.35)	

Haemoglobin level	Normal	13 (65)	72 (62)	1	0.58	15 (60)	70 (62)	1	0.61
	Anaemic	7 (35)	45 (38)	0.74 (0.25–2.16)		10 (40)	42 (38)	1.26 (0.51–3.16)	

Education	None/primary	8 (40)	65 (56)	1	0.20	13 (52)	60 (54)	1	0.93
	Senior/tertiary	12 (60)	52 (44)	1.97 (0.70–5.51)		12 (48)	52 (46)	0.96 (0.39–2.38)	

Household socioeconomic status^b^	Most poor	9 (47)	60 (53)	1	0.90	11 (46)	58 (53)	1	0.47
	Least poor	10 (53)	54 (47)	0.93 (0.33–2.63)		13 (54)	51 (47)	1.40 (0.56–3.54)	

HIV Status	Negative	18 (90)	104 (89)	1	0.97	21 (84)	101 (90)	1	0.73
	Positive	2 (10)	13 (11)	0.97 (0.18–5.08)		4 (16)	11 (10)	1.28 (0.32–5.17)	

Malaria parasites	No	19 (95)	105 (90)	1	0.56	24 (96)	100 (89)	1	0.31
	Yes	1 (5)	12 (10)	0.53 (0.06–4.52)		1 (4)	12 (11)	0.34 (0.41–2.77)	

Any worm	No	11 (55)	29 (25)	1	0.04	7 (28)	33 (30)	1	0.64
	Yes	9 (45)	87 (75)	0.32 (0.11–0.94)		18 (72)	78 (70)	1.29 (0.44–3.73)	

Hook worm infestation	No	16 (80)	55 (47)	1	0.02	15 (60)	56 (50)	1	0.43
	Yes	4 (20)	62 (53)	0.24 (0.07–0.81)		10 (40)	56 (50)	0.68 (0.27–1.74)	

*Mansonella perstans*	No	18 (90)	88 (75)	1	0.27	21 (84)	85 (76)	1	0.37
	Yes	2 (10)	29 (25)	0.42 (0.09–1.97)		4 (16)	27 (24)	0.58 (0.18–1.91)	

*Schistosoma mansoni* infestation	No	15 (75)	95 (81)	1	0.64	19 (76)	91 (81)	1	0.46
	Yes	5 (25)	22 (19)	1.36 (0.42–4.36)		6 (24)	21 (19)	1.49 (0.52–4.33)	

Skin prick test result	Negative	6 (30)	80 (68)	1	0.007	14 (56)	72 (64)	1	0.29
	Positive	14 (70)	37 (32)	4.35 (1.49–12.71)		11 (44)	40 (36)	1.63 (0.65–4.09)	

a Adjusted for age, haemoglobin level, education and household socioeconomic status.

b Household socioeconomic status derived using a score comprised of building materials, number of rooms and items collectively owned (Muhangi et al., 2007).

Worm, malaria and HIV infection status of the women was determined at the time they were enrolled into the EMABS trial (median 2 years, interquartile range 1.6–2.3 years prior to skin prick testing). The percentage of women who had been found to have HIV during pregnancy was similar in all groups (Table 2). The percentage of women with malaria parasites was lower among women with history of asthma or eczema. However, having at least one type of worm infection was less frequent in those with a history of asthma than those without such history and this was statistically significant. There was no difference in the proportion with worms between those with and without history of eczema. The inverse association was particularly marked for history of asthma and hookworm (this was statistically significant) and history of asthma and *M. perstans* (but this was not statistically significant). There was also a negative association between history of eczema and hookworm, and with *M. perstans* but neither was statistically significant. A positive skin prick test was strongly associated with a history of asthma but not history of eczema.

The inverse associations between history of asthma or history of eczema and hookworm were only present in women who had a positive skin prick test [adjusted odds ratio (AOR) 0.26, 95% CI 0.10–0.70, *P* = 0.007 vs. AOR 1.09, 95% CI 0.02–4.15, *P* = 0.89].

### 3.3 Estimates of overall prevalence of skin prick reactivity

The overall prevalence of skin prick test reactivity was calculated in the reconstituted population, described above, with the following results: 30.7% for any allergen, 10.9% for *B. tropicalis*, 16.8% for *Dermatophagoides* mix and 15.8% for cockroach. This reconstituted population was found to be comparable to the general EMABS population in terms of all background characteristics and infection status (results not shown).

### 3.4 Factors associated with a positive skin prick test

Associations between skin prick test and maternal education and most infections were weak, but there was a marked inverse association with infection with *M. perstans* (Table 3), which was not explained by measured potential confounders.

**Table 3:** Risk factors associated with skin prick test reactivity among women in Entebbe, Uganda

Characteristic		Proportion skin prick test positive *n*/*n* (%)	Crude odds ratio	Adjusted odds ratio^a^ (95% CI)	*P-*value
Education	None/primary	15/55 (27)	1	1	0.54
	Senior/tertiary	16/46 (35)	1.42	1.37 (0.50–3.78)	

Household socioeconomic status	Most poor	21/54 (39)	1	1	0.02
	Least poor	10/45 (22)	0.45	0.28 (0.09–0.82)	

HIV status	Negative	26/89 (29)	1	1	0.50
	Positive	5/12 (42)	1.73	1.69 (0.37–7.73)	

Malaria parasites	No	30/89 (34)	1	1	0.22
	Yes	1/12 (8)	0.18	0.23 (0.02–2.49)	

Hookworm infestation	No	15/45 (33)	1	1	0.70
	Yes	16/56 (29)	0.80	0.81 (0.28–2.34)	

*Mansonella perstans*	No	29/75 (39)	1	1	0.02
	Yes	2/26 (8)	0.13	0.14 (0.03–0.69)	

*S. mansoni* infestation	No	26/83 (31)	1	1	0.93
	Yes	5/18 (28)	0.84	1.06 (0.27–4.10)	

a Adjusted for age, education and socioeconomic status.

## 4 Discussion

Based on the results of this study, we estimated that 30.7% of women in our trial population were atopic. House dust mites and cockroach were the commonest causes of sensitization to allergens among participating women in Entebbe, Uganda. Sensitization to dog, cat, grass, pollen and mould allergens was rare. Women with a reported history of asthma, but not eczema, were more likely to show skin prick test reactivity than those with no such history. Women with *M. perstans* infection had significantly lower prevalence of atopy, and hookworm was strongly associated with reduced odds of a history of asthma, but this was not significant for a history of eczema and the effect was restricted to women with atopic sensitization.

The prevalence of atopy is similar to that which has been found in other African sites (Benzarti et al., 2002, Nyan et al., 2001, Shaheen et al., 1996). The observation that house dust mites were the commonest cause of sensitization accords with patterns observed elsewhere in Africa (Warrell et al., 1975), in Asia (Chew et al., 1999, Leung et al., 1997) and in South American countries (Montealegre et al., 1997). This contrasts with Europe (Eriksson and Holmen, 1996) and Arabian Gulf countries (Bener et al., 2002), where pollen and cats have been observed to be equally important causes of sensitization. It was interesting to note that only one woman was sensitized to weed pollen, none to moulds and very few to cat epithelia, yet these allergens are very common in this environment. These results emphasize that sensitization patterns vary between regions of the world. In order to understand allergic sensitization, each region needs to identify its own pattern; further, these patterns may change over time (Eriksson and Holmen, 1996).

This study was among only women; it is possible that immune responses in men may be different. These are initial results that would not necessarily be the same for the general Entebbe population.

There was a strong association between reported history of asthma and prevalence of atopic sensitization, suggesting good internal validity for this measure. This was reassuring since the reported history of asthma was not validated. However, the prevalence of positive skin prick test responses among women with reported history of asthma in this study was relatively low compared to previous reports of responses among asthmatics elsewhere (Chew et al., 1999, Montealegre et al., 1997). This could reflect a different balance of atopic and non-atopic asthma, or atopy to an untested allergen that contributes to asthma in this population. The lower prevalence of atopic sensitization among women with a history of eczema compared to those with a history of asthma is an indication that the two disease entities are different; however, it could also be due to misclassification since eczema did not have a name in the local language and is not a well recognized condition in this setting.

The worm infection status of the women was established at least 1 year before skin prick testing was carried out, and as the women received helminth treatment in the interval, the status at the time of skin prick testing was unknown for all worms except for *M. perstans*. The lack of association with other worms described must therefore be regarded as lack of association with a history of infection, rather than with current infections. *Mansonella perstans*, on the other hand, is not susceptible to the treatments used in this study, and it is unlikely that women received treatment outside the trial as this infection is largely non-pathogenic: usually no treatment is indicated. Adult worms are long-lived, and it is probable that the infection status of many of the women remained unchanged between pregnancy and skin prick testing.

Despite the limitations of the study, our findings are in keeping with several earlier studies suggesting inverse associations between chronic worm infections and allergy and atopy in regions of high prevalence of worm infections (Nyan et al., 2001; van den Biggelaar et al., 2001). For hookworm, we found an inverse association with allergic disease, but only a weak inverse association of atopy with worm infection about 1 year earlier. This could occur if any ‘protective’ effect of hookworm against atopic responses had been removed by treatment of hookworm, and reinfection with hookworm prior to skin prick testing had been limited. Previous studies in school children have produced conflicting results with regard to the effects of deworming on skin prick test responses (Cooper et al., 2006; van den Biggelaar et al., 2004). On the other hand, the strong inverse association between the untreated worm, *M. perstans*, and skin prick test responses suggests that persistent infection may be important. To our knowledge, although much has been written regarding associations between geohelminths (such as hookworm) and atopy, this is the first report of an inverse association with *M. perstans*. This filarial worm is widely distributed in Africa, Central and South America, and the Caribbean. Adult worms live in serous body cavities such as the peritoneum, and reproduce by production of microfilariae, which circulate in the peripheral blood; transmission is by biting midges (Simonsen, 2003). The potent immunomodulating properties of this species are highlighted by the observation that it is largely non-pathogenic even in the presence of thousands of microfilariae per millilitre of blood.

In conclusion, in Entebbe, Uganda, prevalence of atopy among women was similar to that which has been found in other African settings. House dust mites and cockroach, but not pollen, mould or animal epithelia, are common causes of atopic sensitization. Atopic sensitization was highly associated with asthma but not eczema; *M. perstans* infection appears to reduce the risk of atopy and hookworm the risk of asthma in atopic women.

## Funding

Wellcome Trust as a Masters Training Fellowship held by HM (grant number 074791) and a Wellcome Trust Senior Fellowship held by AME (grant number 064693). Wellcome Trust is a UK registered charity No.210183.

## Conflicts of interest statement

None declared.

## Ethical approval

Ethical approval for this study was obtained from three bodies: Uganda Virus Research Institute Science and Ethics Committee, Entebbe, Uganda; Uganda National Council for Science and Technology, Kampala, Uganda; and London School of Hygiene & Tropical Medicine, UK.

## Authors’ contributions

HM, AME and LCR designed the study protocol; HM, JN, JT and MM were responsible for clinical care of the participants; HM, LM, AME and LCR carried out the analysis and interpretation of these data; HM, AME and LCR drafted the manuscript. All authors read and approved the final manuscript. HM and AME are the guarantors of the paper.

## References

[bib1] Bener A., Safa W., Abdulhalik S., Lestringant G.G. (2002). An analysis of skin prick test reactions in asthmatics
in a hot climate and desert environment. Allerg. Immunol..

[bib2] Benzarti M., Mezghani S., Jarray M., Garrouche A., Khirouni S., Klabi N. (2002). Skin test reactivity to seven aeroallergens in a
Sousse area population sample. Tunis Med..

[bib3] Chew F.T., Lim S.H., Goh D.Y., Lee B.W. (1999). Sensitization to local dust-mite fauna in
Singapore. Allergy.

[bib4] Cooper P.J., Chico M.E., Bland M., Griffin G.E., Nutman T.B. (2003). Allergic symptoms, atopy, and geohelminth infections
in a rural area of Equador. Am. J. Respir. Crit. Care Med..

[bib5] Cooper P.J., Chico M.E., Vaca M.G., Moncavo A.L., Bland J.M., Mafla E., Sanchez F., Rodrigues L.C., Strachan D.P., Griffin G.E. (2006). Effect of albendazole treatments on the prevalence of
atopy in children living in communities endemic for geohelminth
parasites: a cluster-randomised trial. Lancet.

[bib6] Elliott A.M., Mpairwe H., Quigley M., Nampijja M., Muhangi L., Owek-Onyee J., Muwanga M., Ndibazza J., Whitworth J.A. (2005). Helminth infection during pregnancy and development of
infantile eczema. JAMA.

[bib7] Elliott A.M., Kizza M., Quigley M.A., Ndibazza J., Nampijja M., Muhangi L., Morison L., Namujju P.B., Muwanga M., Kabatereine N., Whitworth J.A. (2007). The impact of helminths on the response to
immunisation and on the incidence of infection and disease in
childhood in Uganda: design of a randomised, double-blind,
placebo-controlled, factorial trial of de-worming interventions
delivered in pregnancy and early childhood
[ISRCTN32849447]. Clin. Trials.

[bib8] Eriksson N.E., Holmen A. (1996). Skin prick tests with standardized extracts of
inhalant allergens in 7099 adult patients with asthma or
rhinitis: cross-sensitizations and relationships to age, sex,
month of birth and year of testing. J. Investig. Allergol. Clin.
Immunol..

[bib9] Keeley D.J., Neill P., Gallivan S. (1991). Comparision of prevalence of reversible airways
obstruction in rural and urban Zimbabwean
children. Thorax.

[bib10] Leung R., Ho P., Lam C.W., Lai C.K. (1997). Sensitization to inhaled allergens as a risk factor
for asthma and allergic diseases in Chinese
population. J. Allergy Clin. Immunol..

[bib11] Malhotra I., Ouma J., Wamachi A., Kioko J., Mungai P., Omollo A., Elson L., Koech D., Kazura J.W., King C.L. (1997). In utero exposure to helminth and mycobacterial
antigens generates cytokine responses similar to that observed
in adults. J. Clin. Invest..

[bib12] Montealegre F., Quinones C., Michelen V., Bayona M., Fernandez-Caldas E., Vazques O., Colon F., Chardon D., Garcia M. (1997). Prevalence of skin reactions to aeroallergens in
asthmatics of Puerto Rico. P. R. Health Sci. J..

[bib13] Muhangi L., Woodburn P., Omara M., Omoding N., Kizito D., Mpairwe H., Nabulime J., Ameke C., Morison L.A., Elliott A.M. (2007). Associations between mild-moderate anaemia in
pregnancy and helminth, malaria and HIV infection in Entebbe,
Uganda. Trans. R. Soc. Trop. Med. Hyg..

[bib14] Ng’ang’a L.W., Odiambo J.A., Mungai M.W., Gicheha C.M., Nderitu P., Maingi B., Macklem P.T., Becklake M.R. (1998). Prevalence of exercise induced bronchospasm in Kenyan
school children: an urban-rural comparison. Thorax.

[bib15] Nyan O.A., Walraven G.E.L., Banya W.A.S., Milligan P., Van der Sande M., Cessay S.M., Del Prete G., McAdam K.P. (2001). Atopy, intestinal helminth infection and total serum
IgE in rural and urban adult Gambian communities. Clin. Exp. Allergy.

[bib16] Shaheen S.O., Aaby P., Hall A.J., Barker D.J., Heyes C.B., Shiell A.W., Goudiaby A. (1996). Measles and atopy in Guinea-Bissau. Lancet.

[bib17] Simonsen P.E., Cook G.C., Zumla A. (2003). Manson's Tropical Diseases.

[bib18] van den Biggelaar A.H.J., van Ree R., Rodrigues L., Lell B., Deelder A.M., Kremsner P.G., Yazdanbakhsh M. (2000). Decreased atopy in children infested with Schistosoma
haematobium: a role for parasite-induced
interleukin-10. Lancet.

[bib19] van den Biggelaar A.H., Lopuhaa C., van Ree R., van der Zee J.S., Jans J., Hoek A., Migombet B., Borrmann S., Luckner D., Kremsner P.G., Yazdanbakhsh M. (2001). The prevalence of parasite infestation and house dust
mite sensitization in Gabonese schoolchildren. Int. Arch. Allergy Immunol..

[bib20] van den Biggelaar A.H., Rodrigues L.C., van Ree R., van der Zee J.S., Hoeksma-Kruize Y.C., Souverijn J.H., Missinou M.A., Borrmann S., Kremsner P.G., Yazdanbakhsh M. (2004). Long-term treatment of intestinal helminths increases
mite skin-test reactivity in Gabonese
schoolchildren. J. Infect. Dis..

[bib21] Van Niekerk C.H., Weinberg E.G., Shore S.C., Heese H., Van Schalkwyk D.J. (1979). Prevalence of asthma: a comparative study of urban and
rural Xhosa children. Clin. Allergy.

[bib22] Warrell D.A., Fawcett I.W., Harrison D.W., Agamah A.J., Ibu J.O., Pope H.M., Maberly D.J. (1975). Bronchial asthma in the Nigerian Savanna
region. Q. J. Med..

[bib23] Yemaneberhan H., Bekele Z., Venn A., Lewis S., Parry E., Britton J. (1997). Prevalence of wheeze and asthma and relation to atopy
in urban and rural Ethiopia. Lancet.

